# Evolution of ribozymes in the presence of a mineral surface

**DOI:** 10.1261/rna.057703.116

**Published:** 2016-12

**Authors:** James D. Stephenson, Milena Popović, Thomas F. Bristow, Mark A. Ditzler

**Affiliations:** 1 NASA Postdoctoral Program, NASA Ames Research Center, Moffett Field, California 94035, USA; 2 Space Science and Astrobiology Division, Exobiology Branch, NASA Ames Research Center, Moffett Field, California 94035, USA; 3 Blue Marble Space Institute of Science, Seattle, Washington 98145, USA

**Keywords:** clay, in vitro evolution, mineral, origin of life, ribozyme

## Abstract

Mineral surfaces are often proposed as the sites of critical processes in the emergence of life. Clay minerals in particular are thought to play significant roles in the origin of life including polymerizing, concentrating, organizing, and protecting biopolymers. In these scenarios, the impact of minerals on biopolymer folding is expected to influence evolutionary processes. These processes include both the initial emergence of functional structures in the presence of the mineral and the subsequent transition away from the mineral-associated niche. The initial evolution of function depends upon the number and distribution of sequences capable of functioning in the presence of the mineral, and the transition to new environments depends upon the overlap between sequences that evolve on the mineral surface and sequences that can perform the same functions in the mineral's absence. To examine these processes, we evolved self-cleaving ribozymes in vitro in the presence or absence of Na-saturated montmorillonite clay mineral particles. Starting from a shared population of random sequences, RNA populations were evolved in parallel, along separate evolutionary trajectories. Comparative sequence analysis and activity assays show that the impact of this clay mineral on functional structure selection was minimal; it neither prevented common structures from emerging, nor did it promote the emergence of new structures. This suggests that montmorillonite does not improve RNA's ability to evolve functional structures; however, it also suggests that RNAs that do evolve in contact with montmorillonite retain the same structures in mineral-free environments, potentially facilitating an evolutionary transition away from a mineral-associated niche.

## INTRODUCTION

Interactions between minerals and organic molecules likely played a role in the emergence of life on the early Earth and perhaps even play(ed) a role in the emergence of life on other planets. Mineral surfaces can support several processes that may be exploited by emerging life (Hazen and Sverjensky 2010) including the selective sorption (Franchi et al. 2003), concentration, protection (Biondi et al. 2007b), organization (Hanczyc et al. 2003; Konnyu et al. 2015; Shay et al. 2015), and chemical transformation (Huang and Ferris 2006) of organic molecules. Additionally, similarities between some bioinorganic structures and mineral surfaces suggest that metabolic functions in emerging life occurred on mineral surfaces (Nitschke et al. 2013). It is therefore important to address the role of inorganic structures when considering the processes involved in the origin(s) and early evolution of life.

Clay minerals are among those predicted to be present in prebiotic environments, including the early Earth, where they have been proposed to facilitate the transition from abiotic chemistry to biology. Water on the early Earth (Mojzsis et al. 2001) would have weathered basaltic rocks and generated several different clay mineral species (Hazen et al. 2013). While direct evidence of clay minerals on the early Earth has been lost due to geological cycling, the presence of 3.5 billion year old clay minerals on Mars (Bristow and Milliken 2011) supports their predicted presence on the early Earth and other potentially prebiotic environments. With their small particle size and typically flattened plate-like crystallites, even small proportions of clay minerals in rocks and sediments provide the majority of mineral surface area available for reactions with organic compounds (Ransom et al. 1998). Interaction between organic molecules and clay minerals on the early Earth (or similar habitable planets) is therefore likely and may play a significant role in the emergence of life.

Among the potential interactions between organics and clay minerals, those involving nucleotides and nucleic acids are of particular interest given the central role of RNA in contemporary biology, and evidence of an even greater role for RNA in early life (Benner et al. 2012; Robertson and Joyce 2012). Montmorillonite clay minerals have been shown to bind RNA (Franchi et al. 2003), act as a scaffold to facilitate the formation of RNA from activated monomers (Huang and Ferris 2006; Joshi et al. 2009), and support formation of RNA encapsulating vesicles (Hanczyc et al. 2003, 2007). At least two biologically derived, functional RNA structures (hammerhead and hairpin ribozymes) remain catalytically active in the presence of montmorillonite clay (Biondi et al. 2007a,b); however, it is unclear whether these two structures from contemporary biology are representative of ribozymes in general. Interaction with montmorillonite affects the activity of certain hammerhead ribozymes (Biondi et al. 2007a), and molecular dynamics simulations indicate altered folding pathways for RNA through interaction with montmorillonite (Swadling et al. 2010, 2015). Based on these observations, we predicted that montmorillonite would both interfere with the folding of certain functional structures and stabilize other structures that cannot properly fold without an inorganic scaffold. Through their impact on RNA folding, clay minerals could dramatically alter the distribution of functional RNAs within sequence space, possibly presenting unique opportunities and challenges for nascent life. If clay minerals can support a wider variety of functional structures, then this could make it easier for RNA-based life to emerge in association with a mineral surface; however, if populations evolving in the presence of clay are sufficiently depleted in RNAs that can function in the absence of the mineral surface, this could represent a major challenge in transitioning away from a mineral-associated, initial niche. Our experiments address these possibilities.

To understand broadly how RNA's potential to adopt functional structures can be influenced by the presence of a mineral surface, we evolved RNA populations in vitro in the presence or absence of Na-saturated montmorillonite. The RNA populations were evolved to catalyze RNA cleavage, a function catalyzed by several different RNA structures present in biology (Hammann et al. 2012) and in in vitro evolved populations (Jayasena and Gold 1997; Tang and Breaker 2000; Salehi-Ashtiani and Szostak 2001; Popović et al. 2015). We recently used this in vitro evolution approach to investigate the impact of pH and ion identity on RNA function (Popović et al. 2015), and showed that some structures that are highly favored in one environment are disfavored in others. In contrast, in the study described here we find that the outcomes of parallel in vitro evolution experiments conducted either in the presence or absence of montmorillonite are strikingly similar. This similarity provides evidence that montmorillonite does not provide an enhanced folding environment, but it does demonstrate the potential for a smooth evolutionary transition from an initial mineral-associated RNA world to environments more like the cellular environments of known, contemporary biology.

## RESULTS

### Ribozymes can be readily evolved through selection of self-cleavage activity in the presence of montmorillonite clay

**FIGURE 1.**
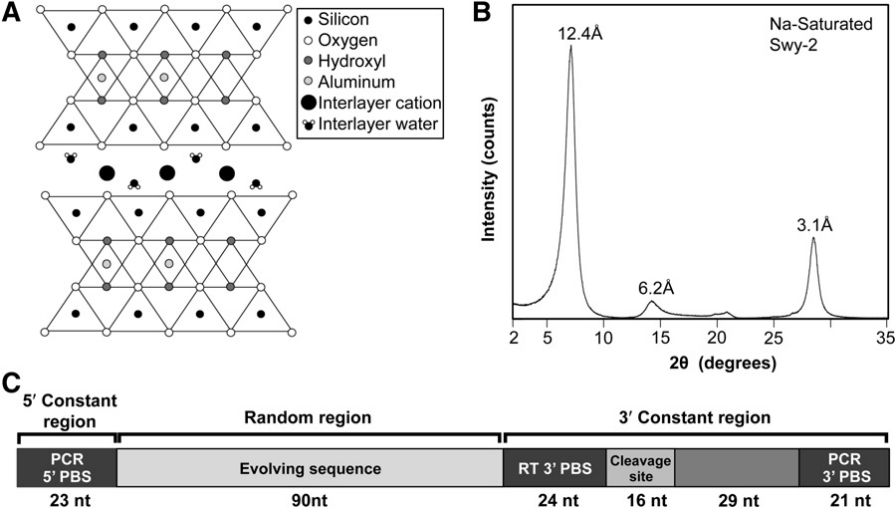
The mineral and RNA used for in vitro evolution. (*A*) 2:1 Phyllosilicate crystal structure of montmorillonite, with layers consisting of Si-bearing tetrahedral sheets sandwiching Al-bearing octahedral sheets. (*B*) X-ray diffraction pattern of the prepared Na-saturated montmorillonite sample in air-dried state confirms the identity and purity of the clay. The intensity of the X-ray reflections are shown as a function of the diffraction angle 2θ along with the corresponding interatomic spacing. (*C*) RNA construct used for in vitro evolution with 90-nt variable sequence, flanked by the 5′ and 3′ constant sequence. The constant sequences contain primer binding sites (PBS) for reverse transcription (RT) and PCR. The 3′ constant sequence contains the 16-nt cleavage site and a 29-nt spacer sequence 3′ of the cleavage site to improve separation between cleaved and uncleaved RNA during electrophoresis.

**FIGURE 2.**
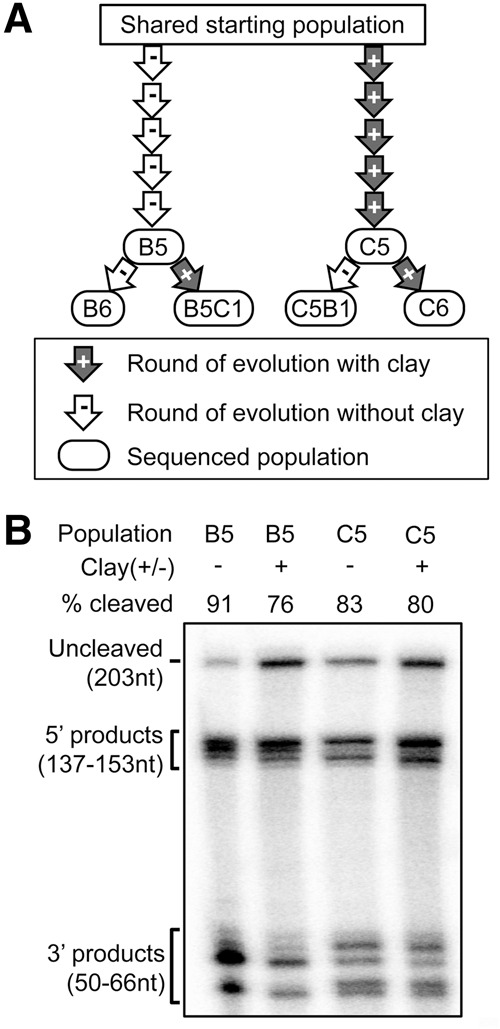
Populations of self-cleaving ribozymes evolved along parallel trajectories. (*A*) Schematic of parallel evolutionary trajectories. Each arrow represents one round of evolution starting from the shared starting population with initial sequence diversity of ∼2 × 10^14^ sequences. Sequenced populations are indicated along with their names. (*B*) Gel showing self-cleavage activity of RNA populations after five rounds of evolution in the absence (B5) or presence (C5) of montmorillonite. Activity is shown for both populations, with [+] and without [−] clay present during the reaction. Ribozymes within the population cleave at different positions in the cleavage sequence, resulting in multiple product bands for each population.

We evolved self-cleaving ribozymes in vitro in the presence or absence of a Na-saturated montmorillonite clay (Fig. 1A,B). The RNA construct used for in vitro evolution was a 203-nucleotide (nt) long RNA with 90 fully random positions, flanked by 5′ and 3′ constant sequences (Fig. 1C). Self-cleaving ribozymes were selected based on their ability to cleave a specific 16-nt target sequence within the 3′ constant sequence. Gel electrophoresis was used to separate active RNA sequences from inactive sequences based on the reduction in length upon self-cleavage. The RNA populations were evolved in parallel, along two separate evolutionary trajectories, starting from a shared, multicopy population of random sequences (Fig. 2A). The selection steps were carried out by first heat denaturing and refolding the populations, either in the presence of 10 mg/mL Na-saturated montmorillonite clay ([+]clay) suspended in a pH 7 buffer with 50 mM NaCl or in the presence of the same buffer without montmorillonite ([−]clay). After refolding, Mg^2+^ was added to a final concentration of 5 mM and the populations were allowed to react for 60 min. As a control for changes in the solution conditions that could occur from exchanges with the clay, the buffer used in the [−]clay selection steps was preincubated with montmorillonite for 60 min and then filtered to remove the clay particles. During the selection, the cleavage reaction was stopped and the RNA was separated from the clay by a 100-fold dilution into a denaturing stripping solution followed by filtration to remove clay particles prior to electrophoresis. Self-cleavage within the populations was apparent during the fifth round of evolution along both the [+]clay and [−]clay trajectories. The populations that emerged from the fifth round (C5 and B5) clearly exhibited self-cleavage activity. Both populations exhibited a similar extent of cleavage in the presence of clay, and for both populations the extent of cleavage is slightly higher in the absence of clay (Fig. 2B). While the extent of cleavage is similar for both populations, the size distribution of the cleavage products shows that the preferred cleavage sites for the two populations are different.

**FIGURE 3.**
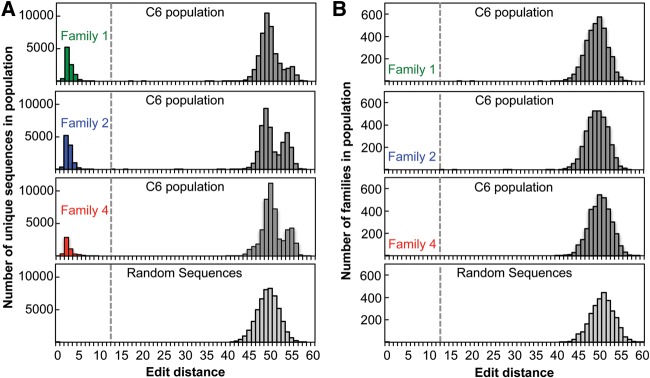
Sequences can be clustered into families of closely related sequences. (*A*) The number of unique sequences within population C6 as a function of edit-distance from the most common sequence from each of families 1, 2, and 4. Sequences to the *left* of the dashed line are within the indicated family. The *bottom* panel shows the edit distance between an arbitrary sequence and a set of randomly generated sequences from a simulated population. (*B*) The number of independent sequence families as a function of edit-distance from the most common sequence from each of families 1, 2, and 4. Edit distances between families are calculated based on the edit distance between the most common sequence within the family. The *bottom* panel shows the edit distance between an arbitrary sequence and a set of randomly generated sequences from a simulated population.

Following in vitro evolution, six populations (B5, B6, B5C1, C5, C6, C5B1) were sequenced using high-throughput sequencing (Fig. 2A). 1.3 × 10^6^ quality-filtered sequence reads were analyzed for each of these populations, which includes between 36,518 and 56,296 unique sequences. For each unique sequence the number of reads was counted. Many sequences are nearly identical to several other sequences in the population. Similar sequences were clustered into sequence families and the number of reads per family was determined. The number of reads for each family in the C6 population was used to assign names to the families based on their rank-order in terms of read abundance. Sequence families are defined such that all members are within 12 edits (substitutions, insertions, or deletions) of the family's most abundant sequence (Fig. 3A). There were between 207 and 3507 sequence families in the populations sequenced. The diversity of sequences within a family likely represents a combination of both mutations to shared parent sequences that arise during the amplification steps of evolution and sequencing errors of a shared sequence. Nearly all sequences that are more than 12 edits apart in sequence space are separated by edit distances between 40 and 55 (Fig. 3A). Those few sequences at edit distances greater than 12 and less than 40 appear to be largely the result of recombination events (Supplemental Fig. S1). Analysis of a simulated population shows that edit distances between random sequences are typically between 40 and 55 (Fig. 3A), indicating that the sequences within a given family are related. Additionally, the edit distances between families in the physical populations are comparable to the edit distances between random sequences in a simulated population (Fig. 3B).

### Shared sequences dominate populations evolved in either the presence or absence of montmorillonite clay

**FIGURE 4.**
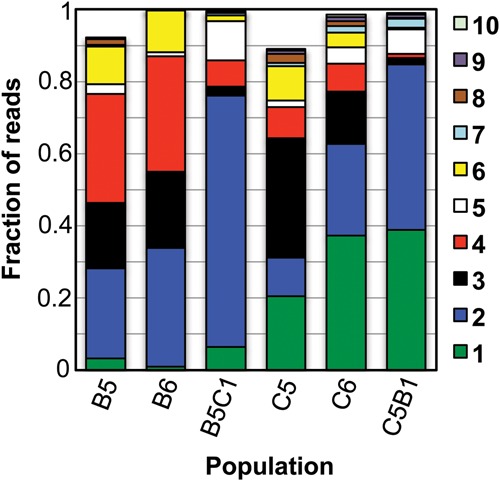
In all populations most of the reads can be clustered into a small number of shared families. The 10 families with the most reads in populations C6 are plotted as stacked bars for each population. For each population, the fraction of the populations’ reads that belong to these 10 families is shown. Each family is color-coded as indicated to the *right* of the graph. Families are numbered based on their abundance in the C6 population.

All six populations are largely composed of the same sequence families (Fig. 4). The 10 families with the most reads in the C6 population account for 98.6% of the reads in that population and >89% of the reads in the remaining populations C5, C5B1, B5, B6, and B5C1. In all cases, the sixth round of evolution resulted in an increase in abundance of the largest sequence families relative to the rest of the population. After the sixth round (both when conditions are held constant and when they are switched), over 98% of sequence reads belong to these 10 shared families.

**FIGURE 5.**
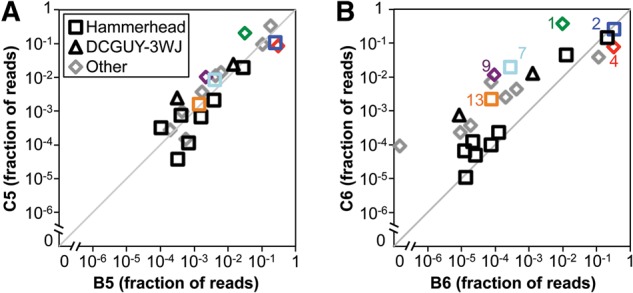
The most abundant sequence families are present at similar abundances in the evolved populations. The fractional read abundances of sequence families within two populations are plotted against each other. The 20 most abundant sequence families within both populations are shown. Families with sequences that contain the hammerhead ribozyme motif are plotted as squares, those containing the DCGUY-3WJ motif are plotted as triangles, and all other families are plotted as diamonds. Families 1, 2, 4, 7, 9, and 13 are labeled and color-coded as in Figure 4. (*A*) Fractional abundances in the [−]clay and [+]clay evolved populations after five rounds of evolution, B5 and C5, respectively. (*B*) Fractional abundances in the [−]clay and [+]clay populations after six rounds of evolution, B6 and C6, respectively.

Populations evolved in the presence or absence of clay are strikingly similar in terms of the identity of sequence families and the associated number of reads (Fig. 5). The differences between populations C5 and B5 (Fig. 5A) are comparable to the differences between a previously generated pair of replicate evolutionary trajectories (Supplemental Fig. S2; Popović et al. 2015). The differences between populations C6 and B6 are slightly greater (Fig. 5B), but their differences are still smaller than those observed in all two-way comparisons between any of our previous nonreplicate populations (Supplemental Fig. S2; Popović et al. 2015). The hammerhead ribozyme motif is common in both the [+]clay and [−]clay trajectories (Fig. 5), and this motif was previously observed in multiple in vitro evolution studies (Tang and Breaker 2000; Salehi-Ashtiani and Szostak 2001; Popović et al. 2015). Additionally, a conserved, three-way junction motif, DCGUY-3WJ (Popović et al. 2015), is also common within both the [−]clay and [+]clay populations (Fig. 5).

**FIGURE 6.**
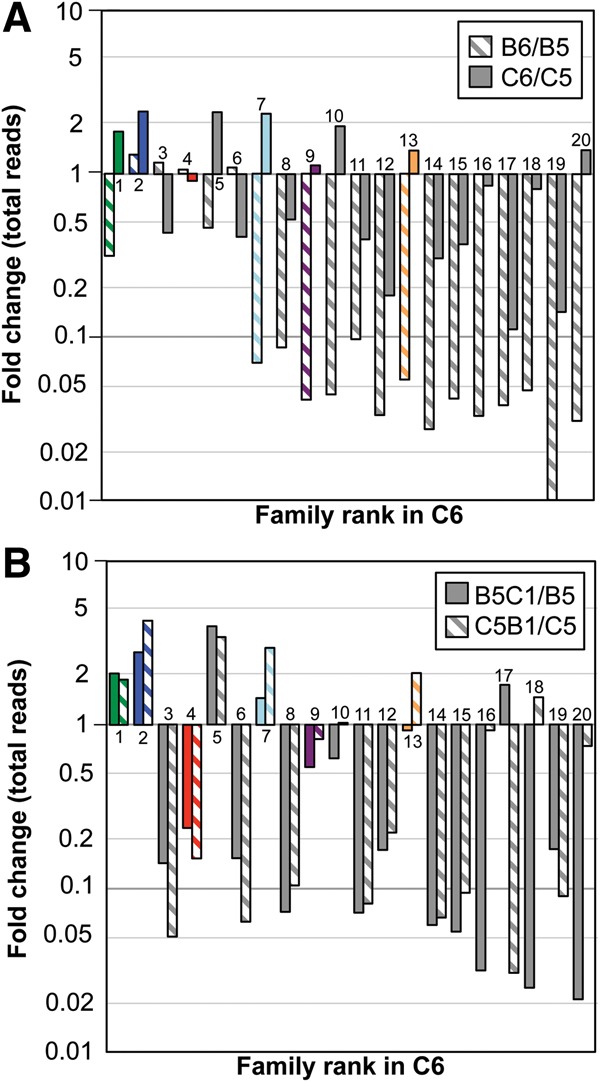
The fold change in the number of reads for families 1–20 during the sixth round of evolution when the conditions are (*A*) kept the same or (*B*) changed relative to the preceding rounds.

To assess the degree to which small differences between the [+]clay and [−]clay populations reflect a response to the presence or absence of clay, we switched conditions in the final round of evolution (Fig. 2A). Both when the populations are kept in the same conditions as the preceding rounds and when the conditions are changed, the number of reads in most families decreases (Fig. 6). An increase in some of the most abundant families shows that they are becoming relatively enriched at the expense of others. For both populations B5 and C5, switching conditions causes the populations to become dominated by Family 2 and the abundance of Family 3 is greatly diminished (Figs. 4, 6B). The direction of change in family abundances is frequently the same whether changing from [−]clay to [+]clay or from [+]clay to [−]clay (Fig. 6B). This suggests that the small differences in the evolved populations reflect small random differences in the starting populations and are not primarily driven by the presence or absence of clay, consistent with the similarity between populations C6 and B6.

### Ribozyme activity is partially inhibited by the presence of clay

**FIGURE 7.**
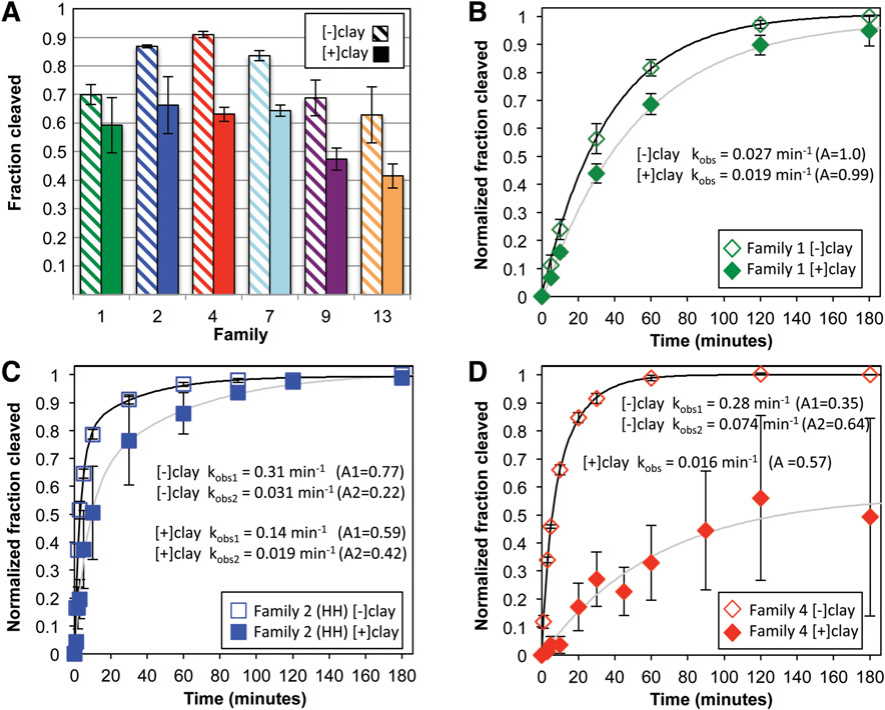
Ribozyme activity assays. (*A*) The fraction of RNA that self-cleaves after incubation under the same conditions used during in vitro evolution for families 1, 2, 4, 7, 9, and 13 in absence (hashed bars) or presence (solid bars) of montmorillonite. Data are represented as mean ± SE. (*B*–*D*) Time courses of self-cleavage activity for families 1,2, and 4 in the absence (open symbols) or presence (filled symbols) of montmorillonite. For time courses, cleavage at *t*_0_ was subtracted and data were normalized to the extent of cleavage at 180 min in the [−]clay conditions. The data are fit with single or double exponential curves (solid lines). Rate constants and amplitudes are indicated in the graphs.

To test the impact of clay on the activity of specific ribozymes within these populations, we assayed the activity of representative sequences (the most abundant sequence within a family) from several families. While sequence comparisons suggest that clay has, at most, a modest impact on relative fitness, we assayed a representative set of sequences for clay-dependent activity. Families 1, 7, 9, and 13 were identified as candidates for sequences with higher activity in clay. They are between 29 and 126 times more abundant in the [+]clay population C6 than in the [−]clay population B6 (Fig. 5B) and increase from round 5 to 6 in the [+]clay trajectory (Fig. 6A). They also decrease in abundance between rounds 5 and 6 in the [−]clay trajectory (Fig. 6A). Family 2 was selected because it grew to dominate the B5C1 population (switch from [−]clay to [+clay]), and a representative from Family 4 was identified as a family that potentially has higher activity without clay. Family 4 is the most overrepresented family (among the 20 most abundant) in B6 relative to C6 (Fig. 5B). Individual representative RNA sequences were prepared and allowed to cleave using the same conditions used for in vitro evolution. Under the selection conditions, all representative ribozymes tested cleaved in both the presence and absence of clay, and all with a lower extent of cleavage in the presence of clay (Fig. 7A). The diminished activity observed in the presence of clay appears to represent a true drop in activity and not an artifact of differential recovery of full-length and cleaved RNA from the clay. When ribozymes were incubated without clay for 1 h and then clay was added for 1 min prior to filtering, the observed cleavage was unchanged relative to the activity without clay (Supplemental Fig. S3).

We further explored the clay dependence of ribozyme activity by measuring cleavage kinetics of individual representatives from families 1, 2, and 4. For kinetic assays, the filtering step, used to remove clay particles prior to electrophoresis during the selection steps, was omitted and samples were loaded directly onto the gels in the stripping solution. Families 1 and 2 have slightly slower rate constants in the presence of clay (Fig. 7B,C) and almost identical amplitudes. Alternatively, Family 4 is more strongly inhibited by clay. The representative from Family 4 was unusual in that, unlike the other five families assayed, it exhibited extensive cleavage during sample preparation, ranging from 25% to 67%. While the remaining uncleaved material is rapidly cleaved in the absence of clay, the rate and magnitude of cleavage are much smaller in the presence of clay (Fig. 7D).

**FIGURE 8.**
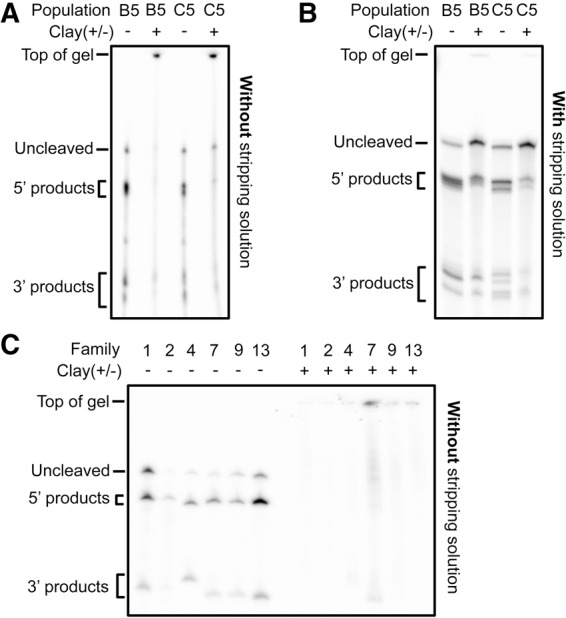
The presence of clay severely impedes the electrophoretic mobility of the evolved RNA when stripping solution is not used to separate RNA from the clay. (*A*) Populations C5 and B5 were incubated for 60 min with or without clay. Samples were diluted fourfold by the addition of buffer with 10% glycerol and then subjected to PAGE. Without the addition of stripping solution, the samples incubated with clay do not fully enter the gel, most of the signal is either immediately below the well at the top of the gel or simply not present. (*B*) Populations C5 and B5 were diluted fourfold by the addition of stripping solution. With the addition of stripping solution, samples incubated with clay enter the gel. The mobility of samples incubated with clay is still slightly retarded relative to samples without clay. (*C*) Individual sequences from families 1, 2, 4, 7, 9, and 13 were incubated with or without clay. The same fraction of the purified transcription product was used for each family so the total signal varies between families. Samples were diluted fourfold by the addition of buffer with 10% glycerol and then subjected to PAGE. Without the addition of stripping solution, the samples incubated with clay do not fully enter the gel. For these sequences, most of the clay-incubated material does not enter the gel at all (the signal is lost) and the little that does enter the gel is mostly at the top.

The similar activities of representative sequences in both the presence and absence of clay and the overall similarity of the populations does not arise from a lack of interaction between the clay particles and the evolved ribozymes. As with the starting population, the evolved populations retain an affinity for montmorillonite (Fig. 8). When [−]clay and [+]clay populations are incubated with clay and then diluted with native buffer, the populations are largely retained within or immediately below the wells during electrophoresis (Fig. 8A). This indicates that the RNA remains associated with the clay particles, which cannot move into the gel. When clay-incubated samples are diluted with stripping solution, they are able to enter the gel, but still have slightly retarded mobility relative to samples without clay (Fig. 8B). Unimpeded mobility can be achieved for clay-incubated samples by dilution into stripping solution followed by filtering (Fig. 2B). Without the addition of stripping solution, the individual representative ribozymes are also retained within or immediately below the wells after clay incubation (Fig. 8C).

## DISCUSSION

RNA's adsorption onto montmorillonite surfaces (Franchi et al. 2003), the reduced activity of at least one biologically derived ribozyme in the presence of montmorillonite (Biondi et al. 2007a), and its impact on RNA folding and dynamics in molecular dynamics simulations (Swadling et al. 2010, 2015) all suggest significantly altered folding landscapes. This impact on folding could make montmorillonite either more or less favorable to RNA-based life. We observe that the presence of montmorillonite did not significantly alter the number or identity of RNAs that can adopt functional structures during in vitro evolution. Additionally, the ribozymes tested here are active in the presence of montmorillonite with only moderate inhibition, and even this limited inhibition may partially reflect indirect effects on folding that arise from the dynamic exchange of ions between solution and montmorillonite particles. The extensive overlap between ribozymes evolving in the presence and absence of a clay mineral surface suggests that the presence of montmorillonite does not provide unique opportunities to adopt functional structures, but it does suggest extensive opportunities for evolutionary transitions from clay mineral associated environments to mineral-independent environments.

The presence of Na-saturated montmorillonite did not significantly impact the evolution of a random RNA population when selecting for self-cleavage. On the population level, whether evolved with or without montmorillonite, there is a similar degree of cleavage after the same number of rounds of evolution (Fig. 2B). At the sequence level, we observe that the same sequence families dominate the majority of the populations in both the [−]clay and [+]clay evolved populations (Figs. 4, 5). Critically, this similarity is not an inevitable consequence of using this starting population or technique, as evidenced by the large differences that were previously observed (Popović et al. 2015) between populations evolved from the same starting population used here and the same partitioning method. The differences between the [+]clay and [−]clay populations are relatively small compared to the differences between our previously evolved self-cleaving ribozyme populations in which pH and ion identity were varied (Fig. 5; Supplemental Fig. S2; Popović et al. 2015). Many of the most abundant sequences that emerged in the [+]clay and [−]clay populations are also among the most abundant sequences present in populations evolved previously, at the same pH, from the same starting population (Supplemental Figs. S2, S4). Additionally, the small differences that are present between the [+]clay and [−]clay populations are not reflected in changes in the populations upon changing the selection condition (Fig. 6), indicating a limited role of montmorillonite in generating those differences. Activity assays indicate that multiple unrelated sequences respond to the presence of montmorillonite similarly; most are moderately inhibited (Fig. 7). Self-cleavage prior to the selections steps could contribute to similarities between evolutionary trajectories, and our activity assays indicate that at least one family (Family 4) can undergo self-cleavage during preparative steps. Yet, multiple lines of evidence indicate that this behavior is not the primary determinant of the similarities we observe. For example, the other ribozymes tested had minimal cleavage prior to incubation. Additionally, Family 4 comprises ≤0.3% of the reads in four prior, independently evolved populations that used this same shared starting population and the same partitioning method, with those populations being selected for self-cleavage activity at pH 5 (Supplemental Fig. 4). In contrast, in the three previous evolution trajectories selected at pH 7, Family 4 is the most abundant family (Supplemental Figs. S2, S4), indicating that the evolutionary success of this sequence family depends more on the pH of the selection steps than on activity during preparative steps.

Multiple structures capable of catalyzing self-cleavage are known to be prevalent within short RNA sequences in random sequence libraries (Jayasena and Gold 1997; Conaty et al. 1999; Tang and Breaker 2000; Salehi-Ashtiani and Szostak 2001) and in biology. At least two common structural motifs that are prevalent when self-cleaving ribozymes are evolved in the presence of montmorillonite are also common in multiple populations evolved in the absence of montmorillonite, the DCGUY-3WJ and hammerhead motifs. The populations evolved here have many abundant sequences that contain the hammerhead ribozyme motif, which was previously observed in multiple populations evolved in the absence of minerals and at similar pH values (Tang and Breaker 2000; Salehi-Ashtiani and Szostak 2001; Popović et al. 2015). The hammerhead ribozyme is the most abundant recurring motif identified in the [+]clay population, with eight of the 20 most abundant families containing this structural motif. The hammerhead is also an abundant recurring motif identified within many biological RNAs (Hammann et al. 2012). The dominant structural motifs in the [+]clay evolved population are therefore clearly compatible with mineral free environments including modern cells.

While clay minerals have several features that could be exploited by emerging life (e.g., their ability to build, concentrate, protect, and organize biomolecules including RNA), our results suggest that expanding the range of functional RNA structures is not one of them. The similarities between the populations evolved here and RNAs evolved previously in vitro and in vivo do, however, suggest the possibility of a smooth evolutionary transition from biomolecules evolving in association with mineral surfaces to evolving in mineral-free protocellular environments. These similarities also increase the confidence with which insights derived from in vitro evolution studies performed without minerals, can be applied to origin of life scenarios involving mineral surfaces (Hanczyc et al. 2003; Briones et al. 2009; Konnyu et al. 2015; Shay et al. 2015). While the presence of montmorillonite had surprisingly little impact on the evolution of ribozymes that catalyze self-cleavage, it remains to be seen if intermolecular functions such as ligand binding or RNA ligation are more strongly impacted by the presence of this mineral surface. Furthermore, other mineral surfaces, even other classes of montmorillonite, vary in their characteristics (Joshi et al. 2009; Swadling et al. 2013) and may have different impacts on RNA evolution.

## MATERIALS AND METHODS

### Preparation of montmorillonite

Wyoming Montmorillonite SWY2, purchased from the clay mineral society was disaggregated in deionized water using a sonic horn and <0.5 µm aggregates separated using a high capacity centrifuge (6 × 1 L) at 2500 rpm with 12-min run times. Organics were removed from clay minerals using multiple treatments of 5% hypochlorite solution, adjusted to pH 7 with HCl, a treatment that is mild enough to not damage the clay. Interlayer cations were exchanged by continuous stirring in a 1 M NaCl solution for an hour, followed by centrifugation, removal of the supernatant solution and replacement with fresh NaCl solution. This process was repeated five times. Unincorporated Na ions were removed from clay minerals by dialyzing with deionized water for several days until conductivity meter readings were <50 mS/m. The Na-saturated montmorillonite was then freeze-dried and stored between experiments in a desiccator. X-ray diffraction patterns of oriented specimens and random powder samples of Na-saturated clay aggregates were obtained in the air-dried state to confirm the identity and purity of the clay. X-ray diffraction patterns were collected on a Rigaku Smartlab XRD.

### Preparation of DNA library and RNA population

A 226 base pair double-stranded DNA template was generated as described (Popović et al. 2015) with a 90-nt random region flanked with constant regions for amplification and size differentiation of the cleaved product. The sense strand DNA sequence is:

*GCCATGTAATACGACTCACTATAGGG***ACACGACGCTCTTC CGATCT**(90N)GGGCATAAGGTATTTAATTCCATACTGGACCC AGTCAGTAGACACAACAAGTTCTTAGACGAGATAATACTACG CTAACACCGCACCAAC; the italicized region is the T7 promoter sequence and the bold region is a PCR and Illumina primer binding site. The underlined region corresponds to the cleavage sequence for the self-cleavage selection.

The DNA library of ∼2 × 10^14^ molecules was transcribed to generate a population of ∼2 × 10^16^ RNA molecules, from which aliquots of ∼10^15^ molecules were taken for each of the two trajectories. Transcription was carried out in transcription buffer (50 mM Tris–HCl pH 7.5, 10 mM NaCl, 30 mM MgCl_2_, 2 mM spermidine, 40 mM DTT) with 5 mM of each NTP, 100 µM blocking oligo and T7 RNA polymerase (Promega) for 15 h at 37°C. The blocking oligo (CTACTGACTGGGTCCAG), which is fully complementary to the cleavage sequence, was included to inhibit undesired self-cleavage during transcription (Salehi-Ashtiani and Szostak 2001; Saksmerprome et al. 2004). RNA was purified and the blocking oligomer was removed through denaturing PAGE. The RNA population was recovered from the gel through electro-elution (Biorad), precipitated by the addition of 1/10th volume 3 M NaOAc pH 5.2 followed immediately by addition of three volumes of 100% ethanol and centrifugation at 18,000*g* for 60 min, and then resuspension with water.

### Evolution of site-specific cleavage in the presence or absence of clay

For each selection step the populations were refolded by heating to 90°C for 3 min and cooling to an ambient temperature over 15 min in a buffer with or without clay (1 µM RNA, 50 mM NaCl, 50 mM MOPS pH 7, and 10 mg/mL of montmorillonite for the [+]clay selections). For the [−]clay selection steps, prior to addition of RNA, the above buffer was preincubated for 60 min with 10 mg/mL sodium exchanged montmorillonite and then filtered through a 150 k MWCO filter (Pierce) to remove clay particles. Removal of clay particles upon filtering was verified by the loss of the characteristic UV absorbance peak of montmorillonite. After filtering, UV absorbance is <1% of the initial suspension and indistinguishable from background. This preincubation step allowed equilibration between the clay and the buffer so that changes to the buffer that could arise from the presence clay would be consistent between the two evolutionary trajectories. The preincubation does not alter the pH of the buffer. After refolding, MgCl_2_ was added to a final concentration of 5 mM Mg^2+^ and the samples incubated for 60 min at ambient temperature (23°C). A 100× volume of stripping solution (10 M urea, and 20 mM EDTA adjusted to pH 10 with NaOH) was added to stop the reaction and dissociate the RNA from the clay. The samples were then filtered through a 150 k MWCO filter to remove the clay particles. The flow-though was ethanol precipitated, resuspended in denaturing loading buffer and subjected to denaturing PAGE (8 M Urea, 6% polyacrylamide, 2 mM EDTA, 89 mM boric acid, 89 mM Tris, pH 8.3). Denaturing PAGE was used to separate the active sequences within the RNA population from inactive full-length RNA. Size standards were run alongside the population during PAGE so that only those sequences that cleave within the defined cleavage sequence were selected. The catalytically active sequences were recovered from the gel through electro-elution (Biorad), precipitated, and resuspended in water. The resuspended sample was then reverse transcribed using ImProm-II reverse transcriptase (Promega), and amplified via PCR using Taq DNA polymerase (Thermo Scientific). Finally, the PCR products were transcribed in vitro to generate the RNA population used in the next round of evolution. This process was repeated for six rounds.

### Sequencing and analysis of evolved populations

In vitro evolved populations were sequenced on an Illumina HiSeq 2500 instrument. The six populations described here were sequences along with 16 additional populations on a single lane. Prior to sequencing, populations were reverse transcribed and PCR amplified with primers that introduced indexing sequences that allowed the multiplexing of multiple populations in a single-sequencing lane. Phusion High-Fidelity DNA polymerase (Thermo Scientific) was used for this PCR step to minimize mutations after the final selection step. All populations were diluted to the same concentration. Approximately 7 million raw sequence reads per population contained information on 100 positions per molecule. Constant sequences on the 3′ end of the sequences reads were removed from the variable regions of 85–93 nt, and raw reads were quality filtered by completely removing all reads in which any position within the variable region has a Phred score of <29 using a custom Python script. For all populations >1.3 million reads remained after quality filtering. For comparative analysis, 1.3 million reads were chosen randomly from each population. Reads were counted, clustered into families of related sequences and compared between populations using FASTAptamer toolkit (Alam et al. 2015) with an edit distance of 12 used to define sequence families. The hammerhead and DCGUY-3WJ motifs were identified using motif descriptors as described (Popović et al. 2015). Simulated populations were generated using a custom Perl script. Simulated populations included the same number of sequences and the same length distribution as the experimental populations.

### Self-cleavage activity assays

RNA was transcribed from DNA templates in the presence of ^32^P α-CTP and the blocking oligo. ^32^P body-labeled ribozymes were purified, refolded, and then incubated. For end point assays of individual sequences, reactions were initiated and stopped in the same way as the selection. For kinetic assays, reactions were initiated as in the selection, but the reactions were stopped by the addition of a 3× volume of stripping solution and run directly on a PAGE gel. Products were separated on 6% PAGE and quantified using ImageQuant software to determine the amount of signal from each band. The extent of cleavage was calculated based on the signal from the bands corresponding to the uncleaved RNA and the 5′ cleavage products, correcting for the difference in the amount of incorporated ^32^P. For kinetic assays, *t*_0_ is defined as the time when Mg^2+^ was added to the reaction. With the exception of Family 4, the extent of cleavage at *t*_0_ is minimal (<5%). Cleavage kinetics were fit to a single *y*(*t*) = *A*(1 − e^(−kobst)^) or double exponential *y*(*t*) = *A*_1_(1 − e^(−*k*obs1*t*)^) + *A*_2_(1 − e^(−*k*obs2*t*)^) using MyCurveFit (MyAssays Ltd.). Prior to the fit, cleavage at *t*_0_ was subtracted and the extent of cleavage was normalized to the maximum extent of cleavage observed in the absence of montmorillonite.

## SUPPLEMENTAL MATERIAL

Supplemental material is available for this article.

## Supplementary Material

Supplemental Material
