# Supplementary material for: Evolution of ribozymes in the presence of a mineral surface
Source: RNA. 2016 Dec;22(12):1893–901. doi: 10.1261/rna.057703.116 (PMC5113209; doi:10.1261/rna.057703.116)
Supplement: Supplemental Material [file supp_22_12_1893__index.html]

Evolution of ribozymes in the presence of a mineral surface — Evolution of ribozymes in the presence of a mineral surface — Supplemental Material 

# Evolution of ribozymes in the presence of a mineral surface

## Supplemental Material

- Supplemental\_Figures\_1-4.pdf
