# Supplementary material for: Evolution of ribozymes in the presence of a mineral surface
Source: RNA. 2016 Dec;22(12):1893–901. doi: 10.1261/rna.057703.116 (PMC5113209; doi:10.1261/rna.057703.116)
Supplement: Supplemental Material [file supp_057703.116_Supplemental_Figures_1-4.pdf]

Supplemental Figure 1

```
Most common in Family 1   CTGCGTGTGACCTAAGTTGCCAGATAGCCCAATTCTCATCTTCAATGATTCTTATCGCGTGTCGGGCTGCTCCGTTTCAATACGGTTCAC (329863)
Edit distance = 17        CTGCGTGTGACCTAAGTTGCCAGATAGCCCAATTCTCATCTTCAATGATTCTTATCTGGTGTGGAGGCATACGTCTCTCACTCAAG (1)
Edit distance = 17        CTGCGTGTGACCTAAGTTGCCAGATAGCCCAATTCTCATCTTCAATGATTCTTATCGAGTCTAGGCTGCTTACACCTCAGTCGATAGGCGAT (1)
Edit distance = 20        CTGCGTGTGACCTAAGTTGCCAGATAGCCCAATTCTCATCTTCAATGATTCTTATCGCGTATTTGGAAGGGGAGAATACGTCTTAACCGT (1)
Edit distance = 35        GGAAGCCAAACCATCAACTTGATCAGATTTGTGCGTTCTGTTGCCATCCGTCTGATGGACTTAGTCCTGCTCCGTTTCAATACGGTTCAC (1)
Edit distance = 36             CAAGCTCTCTCCGTTAGTAGCTTGCGTGCCGAAGAAGATTCGTCTAAACTGTGATATACGTCTGCTCCGTTTCAATACGGTTCAC (2)

Most common in Family 2       GAGGAGTACTAGCTGCCCAATTTTCCGAGACTTGCAGGAATAACTACTCTGAGGAGGCCGCGAAAATCGGCCGAAACGGGCACTCTT(194543)
Edit distance = 13             ATGTGCAATAGTACCTTGATTTTTGAGACTTGCAGGAATAACTACTCTGAGGAGGCCGCGAAAATCGGCCGAAACGGGCACTCAT (1)
Edit distance = 16          GCAGCTTTGCGCAGAACTTTCCCTGACCCGACTTGCAGGAATAACTACTCTGAGGAGGCCGCGAAAATCGGCCGAAACGGGCACTCTT (1)
Edit distance = 28      GGGGGACCGTAAGGTCAATCCGTTCACACAGGGTGCCTTCACGACGTGTGTACTCTGAGGAGGCCGCGAAAATCGGCCGAAACGGGCACTCTT (1)
Edit distance = 29          CGCGAGAATGTCGGGTAATCTGGTATGGACATATGCAAGCACACAGATTTGTAGTAGGCCGCGAAAATCGGCCGAAACGGGCACTCTT (1)

Most common in Family 4   AGTTGGAGGGAGGGAGCATCGGGCTAGTGACGAAAAAGAAGGTCTAGCAAACACAGGAAGGTGCGTCTCTTAACGACGATCTCGAGCCTA (63464)
************************* THERE ARE NO SEQUENCES >9 or <40 edits away ********************************************

Most common in Family 6 GGGGGACCGTAAGGTCAATCCGTTCACACAGGGTGCCTTCACGACGTGTGCGCTGGACCGACATTCGAGTAGCACAGCGTCCACATGAGA(40143)
Edit distance = 24      GGGGGACCGTAAGGTCAATCCGTTCACACAGGGTGCCTTCACGACGTGTGTACTCTGAGGAGGCCGCGAAAATCGGCCGAAACGGGCACTCTT (1)
Edit distance = 32      GGGGGACCGTAAGGTCAATCCGTTCACAGCTGATGAGCGTAAAGCGAAATGGATCTAATGCACTACCCTTGTTCCACGTGTACCATCGA

Most common in Family 7        CAAGCTCTCTCCGTTAGTAGCTTGCGTGCCGAAGAAGATTCGTCTAAACTGTGATACGTCTGCTCTGATGAGCAATGGGCGAAACTT (5112)
Edit distance = 15             CAAGCTCTCTCCGTTAGTAGCTTGCGTGCCGAAGAAGATTCGTCTAAACTGTGATATACGTCTGCTCCGTTTCAATACGGTTCAC (2)

Most common in Family 9  TCACTAGCAAGGTGGTGTTCGCAGTTCAAAAGGTGTTCAGACGCGTCAAGACATGTTGACATTAACGGTTGTCGAAGGAAGATACAGTAT (7961)
************************* THERE ARE NO SEQUENCES >8 or <36 edits away ******************************************

Most common in Family 13  ACTCTGATGATGCGCAAGAAGACCGTCAGCACTGTTCACTTCCAAGATCAGGGAACGGGCAGACTTTGAGCTTACAGCGTGAAACAT (2121)
************************* THERE ARE NO SEQUENCES  >6 or <39 edits away *****************************************

Most common in Family 30  GGAAGCCAAACCATCAACTTGATCAGATTTGTGCGTTCTGTTGCCATCCGTCTGATGGGCTTAGTCCTGCTCCGATTCGATTCGGTAAAC (21)
Edit distance = 6         GGAAGCCAAACCATCAACTTGATCAGATTTGTGCGTTCTGTTGCCATCCGTCTGATGGACTTAGTCCTGCTCCGTTTCAATACGGTTCAC (1)
```

Supplemental Fig. 1. Sequences that are between 12 and 40 edits from some of the most common sequences appear to be the products of recombination during PCR. The most common sequence in families 1, 2, 4, 6, 7, 13 and 30 are given along with sequences that appear to be products of recombination events between these sequences and other sequences in the population or with each other. These sequences have long stretches (>23 nt) that perfectly match the most common sequence within a large family. These long stretches of matching sequence are highlighted. The color of highlighting matches the color-coding used throughout the paper (e.g. sequences that match long portions of the most abundant sequence in family 1 are highlighted in green). The sequence highlighted in gray matches Family 30. The number of sequence reads for each sequence in population C6 is given to the right of each sequence.

Supplemental Figure 2

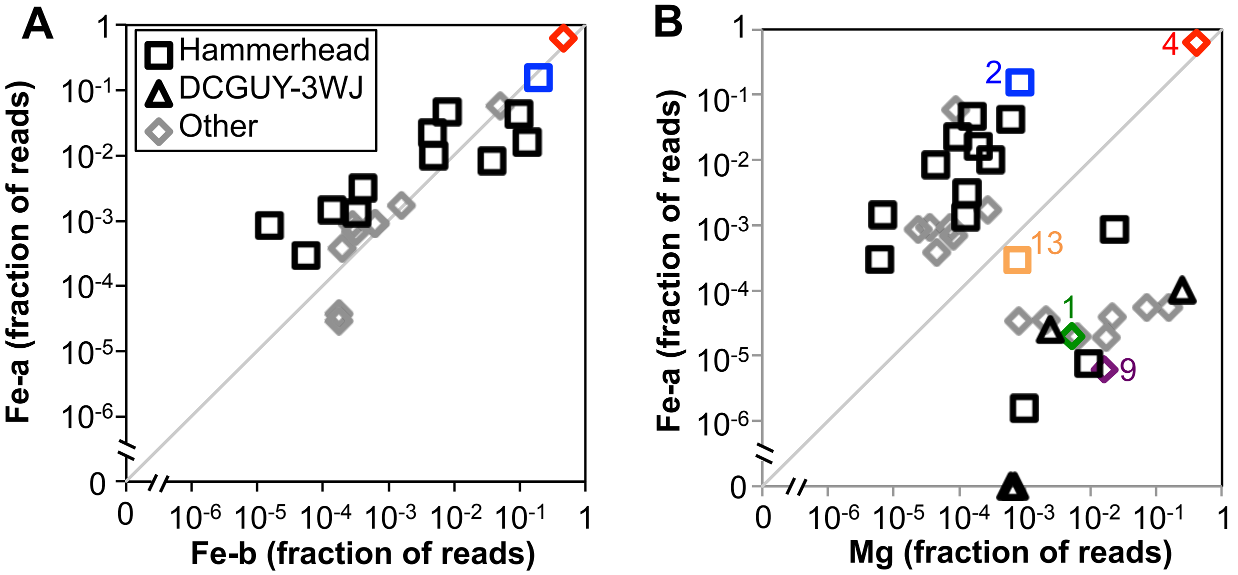

Supplemental Fig. 2. Populations from a previously reported set of evolution experiments were re-analyzed using the same quality cut-off and edit distance cut-off used for the [+]clay and [-]clay populations. The fractional read abundances of sequence families within two populations are plotted against each other. The 20 most abundant sequence families within both populations are shown. Families with sequences that contain the hammerhead ribozyme motif are plotted as squares, those containing the DCGUY-3WJ motif are plotted as triangles, and all other families are plotted as diamonds. Families 1, 2, 4, 9, and 13 are labeled and color-coded as in figure 4. (A) Fractional abundances of two independent evolutionary trajectories evolved using the same selection conditions. These two populations were both evolved by selecting for cleavage in the presences of Fe^{2+} at pH7 for 30 min. (B) Fractional abundances of two independent evolutionary trajectories evolved using different selection conditions. The two populations shown here are the most similar pair among the 6 possible pairwise comparisons of populations evolved under the four different conditions used previously. These two populations were evolved by selecting for cleavage in the presences of either Fe^{2+} at pH7 for 30 min, or in the presence of Mg^{2+} at pH 7 for 30 min.

Supplemental Figure 3

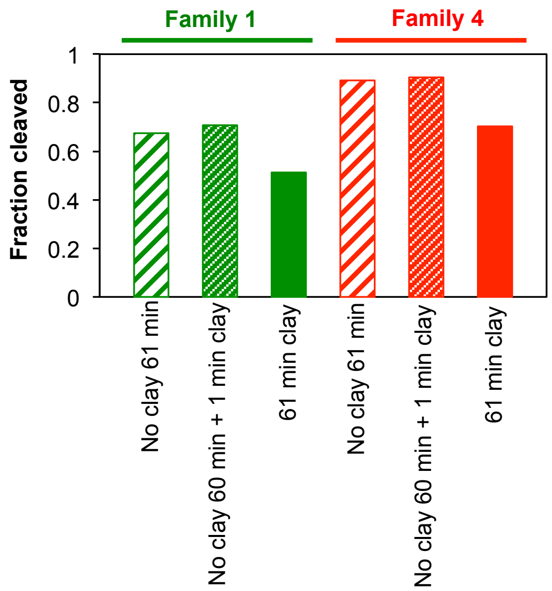

Supplemental Fig. 3. The fraction of RNA cleaved as measured by PAGE following filtering. The extent of cleavage measured for samples incubated without clay for 61 min is the same as the extent of cleavage measured for a sample that has clay added for 1 min immediately prior to stopping the reaction and filtering. For samples with clay present for the entire 61 min, the measured cleavage is lower. Fraction cleaved is shown for the representative sequences from families 1 and 4.

Supplemental Figure 4

Population	Reads from Family 4	pH
Mg.5	0.31%	5
Fe.5	0.05%	5
K.Mg.5	0.13%	5
K.Fe.5	0.06%	5
Mg.7	40.90%	7
Fe.7a	62.42%	7
Fe.7b	46.49%	7
C6	7.7%	7
B6	32.82%	7

Supplemental Fig. 4. The percentage of reads that belong to Family 4 in nine populations is shown along with the pH of the selection step used to evolve the populations. The populations Mg.5, Fe.5, Mg.7, Fe.7a, and Fe.7b are the five populations from the final rounds of evolution reported previously. The percentages above are based on re-analysis using the quality cut-off and edit distance cut-off used here. K.Mg.5 and K.Fe.5 are two additional populations not previously reported. K.Mg.5 and K.Fe.5 populations were evolved for six rounds. The selection steps were carried out in 200 mM KCl at pH 5 for 3.5 hr followed by the addition of 5 mM Mg^{2+}(K.Mg.5) or 5 mM Fe^{2+}(K.Fe.5) with additional 30 min incubation.
